# The Effect of Optimized Ultrafiltration on Perioperative Pulmonary Function During Cardiopulmonary Bypass in Infants Under 10 kg

**DOI:** 10.3389/fped.2021.602034

**Published:** 2021-06-18

**Authors:** Jianhong Niu, Guangdi Zhai, Aibin Zheng, Juanying Zhou, Shengqi Jiang, Jianping Ma

**Affiliations:** Department of Heart, Changzhou Children's Hospital Affiliated to Nantong University, Changzhou, China

**Keywords:** optimized ultrafiltration, 10 kg or less, infants, cardiopulmonary bypass, pulmonary function

## Abstract

**Objective:** This study aims to investigate the effect of optimized ultrafiltration on perioperative electrolytes, acid–base balance, and pulmonary function during cardiopulmonary bypass (CPB) in infants with low body weight (under 10 kg), using traditional balanced ultrafiltration and modified ultrafiltration.

**Methods:** A total of 30 children who underwent surgical correction for congenital heart disease in Changzhou Children's Hospital between January 2018 and December 2019 were randomly divided into two groups. In the treatment group, ultrafiltration pre-treatment was carried out with blood-containing priming fluid prior to CPB. Balanced ultrafiltration was performed during the operation, and optimized and modified ultrafiltration were conducted before closing and extubation. In the control group, traditional balanced ultrafiltration was used during the operation, and a modified ultrafiltration combination was used before closing and extubation. Indexes such as blood gas analysis and electrolytes were measured perioperatively, and pulmonary function was observed.

**Results:** No deaths were reported in either group. The ventilator-assisted breathing time was shorter in the treatment group than in the control group (*P* < 0.05). The indexes of the treatment group were closer than those of the control group to the optimal physiological values. The concentrations of potassium ion (K^+^), lactate (Lac), and blood glucose (Glu) decreased, and there was significant difference between the two groups (*P* < 0.05) at the end of CPB. Hemoglobin (Hb) and hematocrit (HCT) in the treatment group increased (*P* < 0.01). Alveolar-arterial differences for oxygen (A-aDO_2_) and respiratory index (RI) increased significantly in both groups after operation. Children in the treatment group began to recover lung function earlier than children in the control group. Both A-aDO_2_ and RI were lower in the treatment group than in the control group at each time point after operation (*P* < 0.05).

**Conclusion:** Optimizing and modifying the traditional ultrafiltration combination method can effectively shorten the ultrafiltration time, reduce the adverse impacts of the ultrafiltration technique, and improve the lung function of infants after operation.

## Introduction

The amount of priming fluid used in cardiopulmonary bypass (CPB) is significantly higher (with respect to body surface area) in infants than in adults, especially in infants with body weight below 10 kg. Because of infants' low blood volume and the incomplete development of the immune system, vascular endothelial system, lung function, and kidney drainage function, changes in priming fluid composition and the interaction of related factors in the course of CPB have a significant influence on the internal environment of infants, and there is increased likelihood of acute kidney and lung injury (1–3). As such, mitigating these adverse factors for children with a body weight below 10 kg is very important.

This study used a blood ultrafilter to ultrafiltrate the blood-containing priming fluid of infants in the treatment group before CPB operation, and balanced ultrafiltration was then performed during the operation. Before closing and extubation, optimized and modified ultrafiltration was used. The effects of this were monitored and compared with the effects of traditional intraoperative balanced ultrafiltration and modified ultrafiltration before closing and extubation on the control group, in order to observe any adverse impacts of traditional ultrafiltration on the control group and identify reduction of tissue edema or improved lung function in the treatment group.

## Materials and Methods

### Explanation of Terms

#### Ultrafiltration

A pressure-driven membrane separation technology. The aim is to separate macromolecules from small molecules. At a certain pressure, a solvent and a small molecule solute can pass through a special membrane of a particular pore size, while the macromolecular solute cannot pass through; it therefore stays on one side of the membrane and is partially purified.

#### Traditional Ultrafiltration

A modified traditional ultrafiltration technique is used at the end of CPB.

#### Balanced Ultrafiltration

It is the one performed during CPB, which does not modify circulating volume, rather it is supposed to filter out inflammatory mediators. Please refer to these definitions and to these only.

#### Modified Ultrafiltration

Created by Naik. It is the one performed in the OR at the end of CPB, using the very same cannulae in place for surgery. In this process, the inlet end of the ultrafilter is connected with the arterial segment close to the aortic intubation position by a shape tee, the outlet end is connected with the venous return pipeline, and the blood is reinjected into the right atrium. Modified ultrafiltration can be performed during and after CPB.

#### Optimized Ultrafiltration

**Figure 1:**
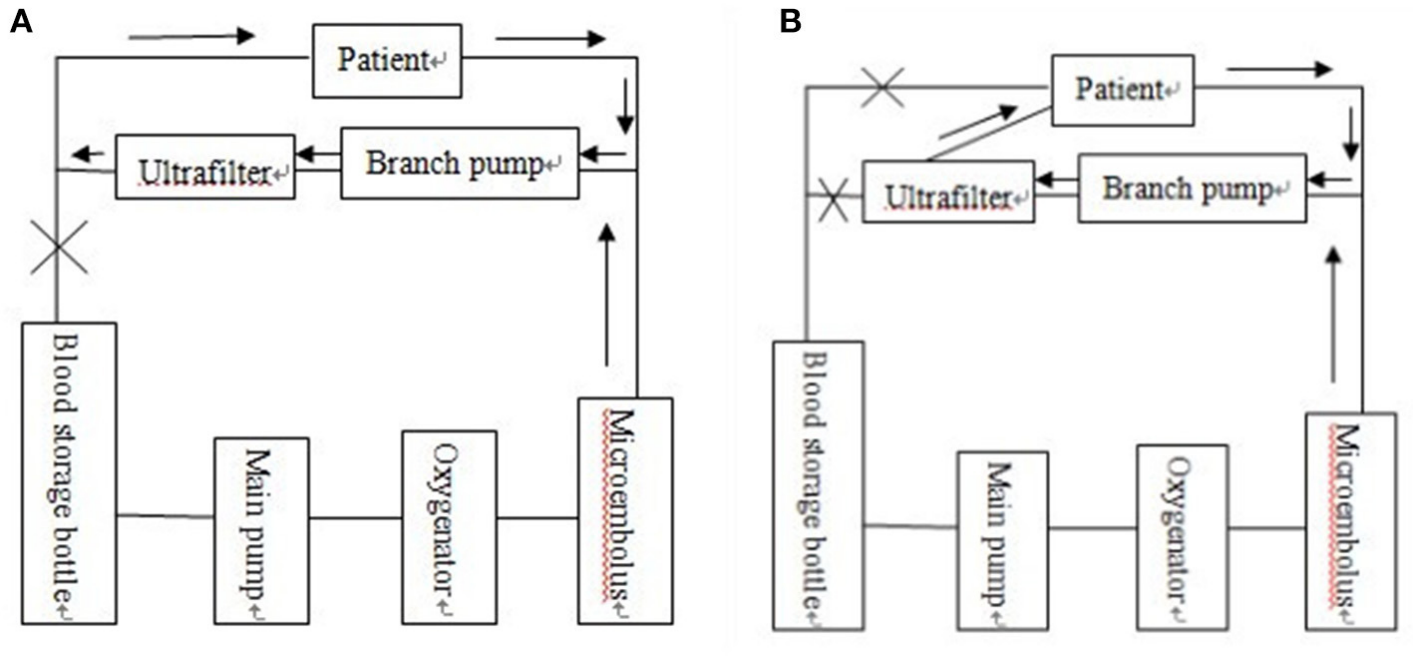
**(A)** Original traditional modified ultrafiltration tube connection mode diagram. **(B)** Modern optimized and modified ultrafiltration tube connection mode diagram.

The combination of three ultrafiltration methods, including preoperative ultrafiltration pretreatment, intraoperative balanced ultrafiltration and modified ultrafiltration before extubation. In this study, the ultrafiltration technology was optimized before the start of CPB, during CPB, and at the end of CPB. On the basis of the optimized ultrafiltration device, a perfusion tube with a diameter of 2 mm was used to connect the right atrium with the ultrafilter. About 70 ml blood in the vena cava pipeline was recovered and the original vena cava circuit was blocked. (Figure 1B, modified on the basis of the original modified ultrafiltration Figure 1A).

### Clinical Data and Grouping

A total of 30 children (body weight ≤ 10 kg) with congenital heart disease treated in Changzhou Children's Hospital between January 2018 and December 2019 were enrolled in the study. A prospective randomized controlled trial was adopted. The first eligible child was divided into treatment group 1, then the second was control group 1; the third was treatment group 2, the fourth was control group 2, and so on. They were divided into a treatment group (n = 15) and a control group (*n* = 15).

In the experimental group, 15 children with congenital heart disease were pretreated with ultrafiltration before CPB, balanced ultrafiltration was performed during operation and modified ultrafiltration was performed before extubation (Optimized ultrafiltration); in the control group, 15 children with congenital heart disease were treated with zero balanced ultrafiltration during operation and modified ultrafiltration before extubation.

The clinical data of the children in the two groups are shown in Table 1. Surgical options were chosen based on the type of disease. These options included repair of a ventricular septal defect (VSD) with patent foramen ovale (PFO), repair of VSD with patent ductus arteriosus (PDA), ligation of ductus arteriosus, repair of atrial septal defect (ASD) with pulmonary stenosis, correction of pulmonary stenosis, and repair of simple atrial and ventricular septal defects. For example, VSD was repaired under cardiopulmonary bypass; ASD was repaired under cardiopulmonary bypass; VSD and PFO were repaired under cardiopulmonary bypass; VSD and PDA were repaired and ligated under cardiopulmonary bypass; ASD with PS was repaired and PS was corrected under cardiopulmonary bypass. The thoracic closed drainage tube is usually placed in the thoracic cavity after the cardiac surgery. According to the amount of postoperative thoracic drainage, it is usually removed after 2–3 days.

**Table 1:** General data of Children in both groups (*n* = 15).

**Clinical data**	**Treatment group**	**Control group**	***P***
Age(m)	8.22 ± 3.23	7.95 ± 2.90	0.419
Weight(kg)	8.56 ± 1.09	8.25 ± 1.23	0.355
Gender (male/female)	7/8	8/7	
Ventricular septal defect with patent foramen ovale	5	6	
Ventricular septal defect with patent ductus arteriosus	1	0	
Ventricular septal defect	5	5	
Atrial septal defect	4	3	
Atrial septal defect with pulmonary stenosis	0	1	

The study was approved by both the ethics committee and the parents of the children.

#### Inclusion Criteria

Children with congenital heart disease, including those requiring repair of VSD with PFO, repair of VSD with PDA, ligation of ductus arteriosus, repair of ASD with pulmonary artery stenosis, correction of pulmonary artery stenosis, and repair of simple atrial and ventricular septal defects were included.

#### Exclusion Criteria

Children with liver and kidney dysfunction, endocrine system diseases, severe pneumonia, heart failure, or coagulation disorders were excluded.

### Methods

#### Anesthesia and Cardiopulmonary Bypass

For anesthesia induction, 2 mg/kg of propofol was used; 20 μg/kg of fentanyl was used as an analgesic, 0.5 mg/kg atracurium was used as a muscle relaxant, and 2% sevoflurane was used for anesthetic maintenance. Before the sternum was split, 10 μg/kg of fentanyl was also administered. The children were treated with a Stockert C5 artificial heart-lung machine, a Capiox Rx05 extracorporeal membrane oxygenation and infantile blood ultrafilter, and an extracorporeal supporting tube produced by Ningbo Feilaer Medical Supplies Co., Ltd. The priming fluid was 500 ml of domestic compound electrolyte injection. The arterial microembolus filter outlet was connected to the blood ultrafilter and its outlet connected to the reflux chamber. The flow rate of the ultrafilter was maintained at 300 ml/min by roller pump.

The optimized and modified ultrafilter had a perfusion tube of about 2 mm in diameter connecting the right atrium and the ultrafilter so that ~70 ml of blood could be recycled in the vena cava tube and the original vena cava circuit blocked. The schematic diagram is shown in Figure 1.

In the treatment group, after the priming fluid was finished, 300 ml of a suspension containing a small number of white and red blood cells, 20 mg of heparin, and 200 ml of plasma were added. Balanced ultrafiltration was then carried out and 500 ml of compound electrolyte injection was gradually added. Approximately 800 ml of liquid was filtered out over the course of 20 min. The temperature of the water tank was maintained at 38°C throughout the procedure and the bank blood was continuously warmed. The ratio of blood flow to ventilation was 1 to 0.5. Oxygen was added to the bank blood and the carbon dioxide removed.

In the control group, 300 ml of a suspension containing a small number of white and red blood cells, 20 mg of heparin, 200 ml of plasma, and 200 ml of compound electrolyte injection were taken for the priming fluid. Self-circulation was carried out for 20 min after priming and exhausting.

Both groups were treated with 5 ml/kg 5% sodium bicarbonate, and all children underwent the CPB operation at room temperature. Myocardial protection was formulated with Del Nido blood-containing cardioplegic solution, with an initial perfusion of 20 ml/kg, followed by 10 ml/kg after 30 min. All children in the treatment group received the priming fluid for ultrafiltration before the operation, balanced ultrafiltration during the operation, and optimized and modified ultrafiltration after the operation; the children in the control group were treated with balanced ultrafiltration during the operation and with traditional modified ultrafiltration after the operation.

#### Detection of Blood Gas and Electrolytes During Cardiopulmonary Bypass

Arterial blood was collected: before CPB after anesthesia induction, 10 min after ascending the aorta, 10 min after the opening of the aorta, and at the end of CPB. All blood samples were analyzed immediately using a GEM Premier 3,000 blood gas analyzer. The testing indexes included blood pH, partial pressure of oxygen (PaO_2_), partial pressure of carbon dioxide (PaCO_2_), base excess (BE), Na+, K+, Ca++, Lac, Glu, Hb, and HCT.

#### Lung Function Test

Arterial blood gas, including arterial PaO_2_ and arterial PaCO_2_, was measured at 2, 6, 12, 24, 48, and 72 h after operation. The alveolar-arterial differences for oxygen (A-aDO_2_) and respiratory index (RI) were calculated using the following formulae:

$$\begin{array}{l} \text{A-}aDO_{2}\left(\text{mmHg}\right)=1^{*}\left(760-47\right)-\text{PaC}{\text{O}}_{2}/\text{R}-\text{Pa}{\text{O}}_{2}\\ \text{ RI}=\text{A-aD}{\text{O}}_{2}/\text{Pa}{\text{O}}_{2}\\ \;(\text{R}=0.85). \end{array}$$

### Statistical Analysis

SPSS 19.0 software was used for the statistical analysis. The results were expressed by mean ± standard deviation (*x* ± s). The paired or grouped *t* test was adopted for normal distribution data. The Wilcoxon signed rank-sum test was used for non-normally distributed data. *P* < 0.05 was considered statistically significant.

## Results

### Clinical Results

No deaths were reported in either group, and all children were discharged smoothly. The difference in CPB time and aortic occlusion time between the two groups was not statistically significant. The ventilator-assisted breathing time of the children in the treatment group was significantly shorter than that of the control group (*P* < 0.05). The post-operative chest drainage volume was lower in the treatment group than in the control group, but there was no statistical significance. See Table 2 for details of clinical results.

**Table 2:** Comparison of intraoperative and postoperative clinical data between the two groups (*x* ± s, *n* = 15).

**Clinical data**	**Treatment group**	**Control group**
CPB time (min)	43.67 ± 8.77	45.50 ± 7.24
Arterial occlusion time (min)	21.83 ± 4.76	22.33 ± 5.69
Ventilator-assisted breathing time (h)	7.23 ± 1.31^*^	17.45 ± 5.93
Chest drainage volume (ml)	140.58 ± 52.96	195.41 ± 73.30

* *Comparison between the treatment group and control group (P < 0.05)*.

### Test Results of Blood Gas and Electrolytes in Children Before and After Cardiopulmonary Bypass

The differences between the treatment group and control group in pH, PaCO_2_, sodium (Na), and calcium (Ca) at each perioperative time point were not statistically significant. The concentrations of K^+^, Lac, and Glu decreased in the treatment group, and were significantly different at the end of CPB from concentrations in the control group (*P* < 0.05). The PO_2_ and BE of the treatment group increased, but the difference compared with the control group was not statistically significant. The Hb and HCT of the treatment group were significantly higher than those of the control group (*P* < 0.01). See Table 3 for further details of these test results.

**Table 3:** Test results of blood gas and electrolytes before and after CPB (x ± s, *n* = 15).

**Indexes**	**Groups**	**Before CPB**	**10 min aortic occlusion 10 min**	**10 min opening of blocked artery 10 min**	**End of CPB**
pH	Treatment group	7.39 ± 0.08	7.43 ± 0.07	7.45 ± 0.06	7.45 ± 0.07
	Control group	7.38 ± 0.04	7.37 ± 0.05	7.44 ± 0.06	7.41 ± 0.04
PO_2_	Treatment group	462.50 ± 119.83	307.50 ± 27.28	287.20 ± 61.13	338.60 ± 146.97
	Control group	388.30 ± 120.57	303.30 ± 62.01	281.40 ± 127.49	304.40 ± 102.17
PCO_2_	Treatment group	37.10 ± 6.90	41.12 ± 6.94	36.17 ± 5.36	36.33 ± 6.22
	Control group	37.46 ± 4.66	42.08 ± 3.34	32.40 ± 11.85	39.50 ± 5.70
BE (mmol/L)	Treatment group	−2.06 ± 1.82	0.96 ± 1.36	1.03 ± 1.70	1.22 ± 2.38
	Control group	−2.63 ± 1.29	−0.18 ± 1.95	0.21 ± 2.23	0.48 ± 1.93
NA^+^ (mmol/L)	Treatment group	138.30 ± 2.16	141.80 ± 2.94	139.30 ± 3.30	142.70 ± 3.30
	Control group	136.80 ± 2.69	140.50 ± 2.59	138.40 ± 2.72	140.10 ± 2.68
K^+^(mmol/L)	Treatment group	3.48 ± 0.30	3.48 ± 0.56	3.99 ± 0.44	3.47 ± 0.42
	Control group	3.61 ± 0.47	3.69 ± 0.63	4.50 ± 0.81	4.15 ± 0.63^*^
Ca^++^(mmol/L)	Treatment group	1.23 ± 0.06	0.75 ± 0.12	0.72 ± 0.59	1.26 ± 0.09
	Control group	1.17 ± 0.25	0.58 ± 0.15	0.79 ± 0.28	1.27 ± 0.10
Lac (mmol/L)	Treatment group	0.79 ± 0.27	1.17 ± 0.21	1.17 ± 0.28	1.06 ± 0.26
	Control group	1.00 ± 0.74	1.18 ± 0.38	1.54 ± 0.44	1.33 ± 0.23^*^
Glu (mmol/L)	Treatment group	4.07 ± 0.69	5.21 ± 0.89	5.46 ± 1.36	5.13 ± 0.98
	Control group	5.48 ± 2.04^*^	6.73 ± 1.78^*^	7.00 ± 1.09^*^	6.20 ± 0.91^*^
HB	Treatment group	9.61 ± 0.96	7.77 ± 0.65	7.97 ± 0.92	12.87 ± 0.63
	Control group	8.93 ± 1.09	7.76 ± 0.84	7.77 ± 0.74	10.54 ± 0.60^**^
HCT	Treatment group	0.28 ± 0.03	0.23 ± 0.02	0.24 ± 0.03	0.38 ± 0.02
	Control group	0.27 ± 0.03	0.23 ± 0.03	0.23 ± 0.02	0.31 ± 0.03^**^

* *Comparison between the treatment group and control group (P < 0.05)*;

** *Comparison between the treatment group and control group (P < 0.01)*.

### Test Results of Pulmonary Function Indexes

In both groups, both A-aDO_2_ and RI increased significantly after operation. However, levels in the treatment group began to decrease and follow a gradually recovering trend 24 h after operation, while those in the control group also showed a downward trend, but later: 48 h after operation. Lung function also tended to recover earlier in the treatment group. At each time point, both A-aDO_2_ and RI were significantly lower in the treatment group than in the control group (*P* < 0.05). See Table 4 for more detail.

**Table 4:** Test results of pulmonary function indexes of children in both groups (*x* ± s, *n* = 15).

**Indexes**	**Groups**	**After operation**					
		**2 h**	**6 h**	**12 h**	**24 h**	**48 h**	**72 h**
A-aDO_2_ (mmHg)	Treatment group	404.90 ± 49.52	509.76 ± 34.63	524.93 ± 20.81	537.06 ± 24.28	534.48 ± 19.43	514.72 ± 18.79
	Control group	556.28 ± 20.81^*^	562.08 ± 18.49^*^	577.33 ± 11.00^*^	579.63 ± 11.71^*^	581.93 ± 8.43^*^	576.93 ± 20.43^*^
RI	Treatment group	1.60 ± 0.49	3.47 ± 1.19	3.77 ± 0.69	4.22 ± 0.96	4.11 ± 0.75	3.92 ± 1.01
	Control group	5.04 ± 0.86^*^	5.59 ± 1.02^*^	6.82 ± 1.10^*^	7.08 ± 1.09^*^	7.54 ± 0.93^*^	7.30 ± 1.59^*^

* *Comparison between the treatment group and control group (P < 0.05)*.

## Discussion

In current practice, in order to maintain proper hematocrit during CPB in infants and young children, a small amount of plasma blood (i.e., red blood cell suspension of low plasma) must be added to the priming fluid. However, the inflammatory mediators and metabolites contained in bank blood, along with low temperature during CPB and many other factors (e.g., hemodynamic changes or contact between the blood and a foreign body), may have adverse effects on cardiopulmonary function (4–6). At present, traditional intraoperative balanced ultrafiltration and modified ultrafiltration combination technology are widely used in cardiac surgery in China. By eliminating excess water and some inflammatory mediators in the body, hematocrit and colloid osmotic pressure can be improved, thereby improving pulmonary function after operation. However, because the method is implemented only after CPB, it often takes about 15 min at a flow rate of 10–15 ml/kg/min to achieve satisfactory results, prolonging the operation time and increasing the negative impacts of the technique (7). The present study investigated the combined application of priming fluid ultrafiltration before CPB, balanced ultrafiltration during operation, and optimized and modified ultrafiltration before closing and extubation in order to observe the perioperative effects of the method on blood gas, electrolytes, and pulmonary function during CPB.

Some studies have found that a small amount of amount of plasma blood in the bank blood is in a state of low temperature (8), low pH, high PCO_2_, high K^+^, and high Glu. Moreover, with prolonged storage time, its cytokines, bradykinin, and other inflammatory mediators increase. The blood *in vivo* also has contact with non-physiological substances during CPB, resulting in changes to the coagulation system, complement system, and various cytokines; this is one of the causes of post-operative organ dysfunction (9). Moreover, the internal environment of infants and young children, especially of children with body weight below 10 kg, is very fragile: because their organs are imperfectly developed, they experience many post-operative complications. The present study shows that the ultrafiltration washing of blood-containing priming fluid can effectively reduce the concentration (10) of tumor necrosis factor alpha (TNF-a) and other inflammatory factors, as well as the levels of Glu, Lac, and K^+^ in the priming fluid.

In the treatment group, the internal environment was closer to the optimal physiological values after stopping CPB and performing optimized and modified ultrafiltration. The concentrations of K^+^, Lac, and Glu decreased in the treatment group, and were significantly different at the end of CPB from concentrations in the control group. The Hb and HCT of the treatment group increased significantly, and were significantly higher than those of the control group.

Previous studies have found that the use of modified ultrafiltration during CPB in low-weight children can significantly improve perioperative and post-operative pulmonary function (11–14). However, as noted above, because the method is only implemented after CPB it prolongs the operation time and increases the adverse impacts of the technique in children, such as hypothermia, air embolus and thrombosis, hemolysis, hypokalemia, hemodynamic instability, and cerebral ischemia. Optimized and modified ultrafiltration can recover about 70 ml of blood in the vena cava duct, which is equivalent to 25% of the whole-body blood volume of a child weighing 3.6 kg. The present study demonstrates increased efficiency of ultrafiltration in the treatment group, which means the whole-body blood circulates faster, thereby shortening the ultrafiltration time.

The postoperative chest drainage volume of the two groups: the control group was larger than the experimental group, as shown in Table 2, but there was no statistical significance; Statistics of blood transfusion after operation or before leaving ICU or discharge: in the control group, 3 blood cells, 2 plasma and 2 cryoprecipitated prothrombin complex were transfused. On the day after operation and the first day after operation, a total of 3.5 units of blood cells, 200 ml of plasma and 6 cryoprecipitated prothrombin complex were transfused; In the experimental group, 4 blood cells were transfused on the first day after operation, a total of 4.0 units. The time of modified ultrafiltration was 15 min at the end of operation in both groups. Results the volume of ultrafiltration fluid in the experimental group was significantly larger than that in the control group, with statistical significance. The protocol is the same as the experimental design.

The post-operative A-aDO_2_ and RI of the treatment group at each time point were significantly lower than those of the control group. Furthermore, the lung function of the treatment group tended to recover earlier. The ventilator-assisted breathing time was significantly shorter in the treatment group than in the control group and the post-operative chest drainage volume was lower than that of the control group, which may be related to a decrease in the concentration of inflammatory mediators in the priming fluid of the treatment group.

The decline of pulmonary function after CPB is significantly positively correlated (15) with the neutrophil and IL-8 levels in bronchoalveolar lavage fluid. The results of this study show that the blood injected into the right atrium through the ultrafiltration perfusion tube during the optimized and modified ultrafiltration had high HCT, high colloid osmotic pressure, high coagulation factor, and low inflammatory factor, which can quickly improve the HCT and shorten the time to the optimal oxygen-carrying capacity in the blood after operation, thereby repaying the tissue oxygen debt more quickly and effectively. Therefore, optimized and modified ultrafiltration is important to reduce the negative effects of traditional modified ultrafiltration technology.

## Conclusion

The combined perioperative application of priming fluid ultrafiltration, balanced ultrafiltration, and optimized and modified ultrafiltration can bring the internal environment of the body closer to the optimal physiological state during CPB. This is expected to effectively shorten the ultrafiltration time from that required by traditional modified ultrafiltration, thus reducing the negative effects of the modified ultrafiltration technology and improving the lung function of infants after operation when CPB surgery is necessary for low-weight infants with congenital heart disease.

## Data Availability Statement

The original contributions presented in the study are included in the article/supplementary material, further inquiries can be directed to the corresponding author/s.

## Ethics Statement

The studies involving human participants were reviewed and approved by Ethics Committee of Changzhou Children's Hospital. Written informed consent to participate in this study was provided by the participants' legal guardian/next of kin. Written informed consent was obtained from the individual(s), and minor(s)' legal guardian/next of kin, for the publication of any potentially identifiable images or data included in this article.

## Author Contributions

JN and GZ conceived the idea, conceptualized the study, and drafted the manuscript. AZ collected the data. JZ analyzed the data. SJ and JN reviewed the manuscript. All authors read and approved the final draft.

## Conflict of Interest

The authors declare that the research was conducted in the absence of any commercial or financial relationships that could be construed as a potential conflict of interest.
